# Quantitative detection of rare interphase chromosome breaks and translocations by high-throughput imaging

**DOI:** 10.1186/s13059-015-0718-x

**Published:** 2015-08-03

**Authors:** Bharat Burman, Tom Misteli, Gianluca Pegoraro

**Affiliations:** Cell Biology of Genomes, National Cancer Institute, National Institutes of Health, Bethesda, MD 20892 USA; Program in Cell, Molecular, and Developmental Biology, Tufts University Sackler School of Biomedical Sciences, Boston, MA 02111 USA; NCI High-Throughput Imaging Facility, National Cancer Institute, National Institutes of Health, Bethesda, MD 20892 USA

## Abstract

We report a method for the sensitive detection of rare chromosome breaks and translocations in interphase cells. HiBA-FISH (High-throughput break-apart FISH) combines high-throughput imaging with the measurement of the spatial separation of FISH probes flanking target genome regions of interest. As proof-of-principle, we apply hiBA-FISH to detect with high sensitivity and specificity rare chromosome breaks and translocations in the anaplastic large cell lymphoma breakpoint regions of *NPM1* and *ALK*. This method complements existing approaches to detect translocations by overcoming the need for precise knowledge of translocation breakpoints and it extends traditional FISH by its quantitative nature.

**Electronic supplementary material:**

The online version of this article (doi:10.1186/s13059-015-0718-x) contains supplementary material, which is available to authorized users.

## Background

Balanced chromosome translocations are among the most common genetic aberrations found in human cancers [1]. Translocations are often causally associated with disease and are frequently used for diagnostic purposes [2, 3]. In clinical practice, translocations are routinely detected by cytogenetic and polymerase chain reaction (PCR)-based methods. PCR is widely used for detecting translocations, however, this approach requires relatively precise knowledge of the break sites. Since translocation breakpoints are often not precisely mapped or may be found over large genomic distances in a given translocation partner, their detection by PCR requires extensive multiplexing of primer sets or use of nested PCR approaches, making them often impractical, especially for routine diagnostic purposes. More recently, genome-wide sequencing approaches have enabled detection of translocations in an unbiased fashion but with considerably reduced sensitivity compared with PCR-based methods [4–6].

A prominent cytological method for detection of translocations is fluorescence in situ hybridization (FISH), which is commonly used in biological and clinical applications. In these approaches, the physical pairing of translocation partners can be detected as the co-localization of FISH probes targeted to the involved translocation genes in metaphase chromosome spreads [7]. Specificity and sensitivity of standard FISH can be greatly increased by the use of break-apart FISH probes consisting of two differentially labeled probes placed upstream and downstream of the putative breakpoint region [8, 9]. Chromosome breakage is indicated by separation of the two probes and, if combined with a third probe targeted towards a putative translocation partner, translocations can be detected by co-localization of a separated break-apart probe with the translocation partner. A major advantage of using break-apart FISH probes over PCR analysis to detect translocations is that precise knowledge of the translocation partner or chromosome breakpoint is not required and probes can be designed so that large regions, up to 500 kb, can be interrogated [7–10].

A major limitation of standard or break-apart FISH is that it requires visual inspection of a large number of cells to detect a sufficient number of chromosome breakage or translocation events, and determining a split signal may be biased by user subjectivity. For practical reasons, the number of cells analyzed by traditional FISH is typically limited to a few hundred and as such FISH is well suited for analysis of cell populations that contain frequent translocations, while detection of rare translocations is frequently prohibitive. In addition, visual inspection of relatively small cell numbers makes it difficult to measure statistically significant differences between biological samples containing low-frequency chromosome breakage and translocation events [7–9].

**Fig. 1:**
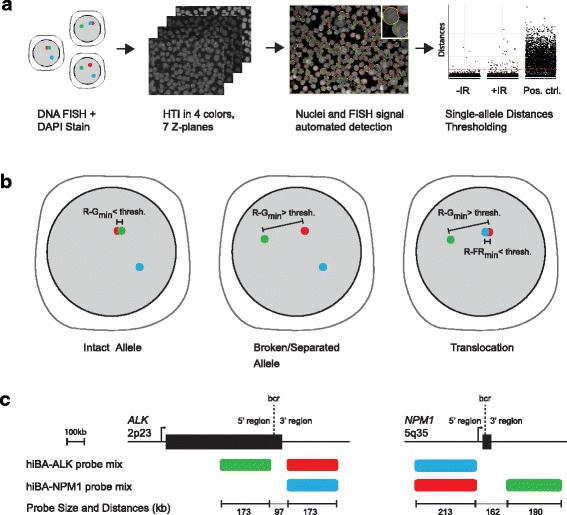
hiBA-FISH outline. **a** hiBA-FISH pipeline. The green, red and blue dots represent FISH signals in fixed interphase cell nuclei. *DAPI* 4',6-diamidino-2-phenylindole, *FISH* fluorescence in situ hybridization, *HTI* high-throughput imaging, *IR* ionizing radiation, *Pos. ctrl.* positive control. **b** Outline of hiBA-FISH event definitions based on the thresholding of relative Euclidean distances of FISH signals in different colors. *R-G*_*min*_ and *R-FR*_*min*_ indicate the per Red signal minimum Red/Green and Red/FarRed distances, respectively. **c** Schematic representation of the size and location of the chromosome breakpoint regions recognized by the two different hiBA-FISH probe sets used in this study. *Bcr* breakpoint cluster region

Considerable progress has recently been made in high-throughput imaging (HTI) and automated image analysis [11–14]. We report here the development of a systematic and unbiased method for the quantitative detection of rare chromosome breakage and translocation events in interphase cells by combining break-apart FISH with HTI. We implement a technique, referred to as hiBA-FISH (high-throughput break-apart FISH) that is based on the detection by HTI of the physical separation in three-dimensional (3D) space of break-apart probes flanking putative translocation breakpoint regions (Fig. 1a). hiBA-FISH consists of fixation of interphase cells on coverslips, followed by DNA FISH using translocation gene-specific break-apart probes. Large image datasets containing thousands of cells per experimental condition are acquired using automated 3D confocal high-throughput microscopy and analyzed using high-content image analysis software to determine the spatial positioning of FISH signals in three separate channels and to calculate distances between them. To detect chromosome breakage and translocation events, FISH signal distance datasets are analyzed using statistical analysis software and frequencies of chromosome breakage and translocation events are measured by establishing distance thresholds for the FISH probes (Fig. 1b). A major advantage of hiBA-FISH is its use of interphase cells, thus alleviating the requirement for metaphase chromosome preparation and enabling highly quantitative determination of breakage and translocation frequencies in a population. As proof-of-principle, we applied hiBA-FISH to measure the number of chromosome breaks at the *NPM1* and *ALK* gene loci and the frequency of the anaplastic large cell lymphoma-specific *NPM1*-*ALK* translocation upon irradiation [15]. We demonstrate sensitive detection of rare chromosome breakage and translocation events by hiBA-FISH.

## Results

### Break-apart probe design

hiBA-FISH is based on the combinatorial use of break-apart probes that flank known or putative translocation breakpoints (Fig. 1b). Several commercial, quality-controlled break-apart probes are readily available and can be used for hiBA-FISH, or break-apart probes can be generated for virtually any region of the genome by incorporation of fluorescent nucleotides into bacterial artificial chromosome (BAC) DNAs using standard nick translation [16]. Suitable BAC DNAs upstream and downstream of the target breakpoints (up to a few hundred kilobases) are readily identified using the University of California, Santa Cruz (UCSC) genome browser. Ideally, BAC DNAs with similar sequence lengths should be selected for the two flanking probes to generate similar FISH signal sizes, although signal size may be influenced by secondary DNA structure and should be optimized by visual inspection of putative probes [16].

When used alone in interphase cells, two-color break-apart probes report on chromosome breakage (Fig. 1b). The two signals of a break-apart probe pair are in proximity at the intact allele (Fig. 1b). Chromosome breakage of the region between the two probes is indicated by separation of the break-apart probes (Fig. 1b). In addition, the combination of a two-color break-apart probe with a third color probe targeted to a translocation partner can be used to identify translocation events, which are detected by the concomitant proximity of a separated break-apart probe with the probe targeted against a known translocation partner (Fig. 1b).

As a model system to develop and test hiBA-FISH, we designed probes for the well-characterized recurrent translocation between the 5’ region upstream of the *NPM1* breakpoint in intron 4 (chromosome 5q35) and the 3’ region downstream of the *ALK* breakpoint in intron 19 (chromosome 2p23) in anaplastic large cell lymphoma (ALCL) [17]. We created two separate three-color probe sets named after the break-apart probes they contain (Fig. 1c). The hiBA-ALK probe set consists of an Alexa488-labeled probe (Green) targeting the 5’ region upstream of the *ALK* breakpoint, an Alexa568-labeled probe targeting the 3’ region downstream of the *ALK* breakpoint, and a Cy5-labeled probe (FarRed) targeting the 5’ region upstream of the *NPM1* breakpoint (Fig. 1c). The hiBA-NPM1 probe set was designed analogously (Fig. 1c).

### Automated hiBA-FISH signal detection

**Fig. 2:**
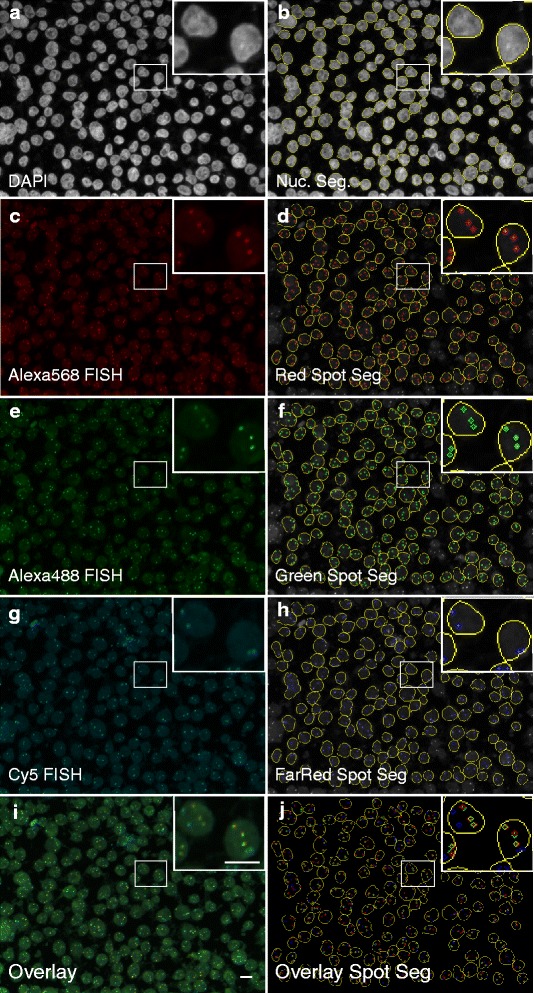
Automated nucleus segmentation and FISH signal detection. **a**, **c**, **e**, **g**, **i** Maximal projections of 40× confocal image z-stacks of Mac2A cells stained with the hiBA-ALK probe set. The overlay represents a composite image of the Green (Alexa488), Red (Alexa568) and FarRed (Cy5) channels. The inset in each panel represents a magnified image of representative Mac2A cells. *DAPI* 4',6-diamidino-2-phenylindole. *Scale bars* 10 μm. **b**, **d**, **f**, **h**, **j** In silico generated images representing the detected nucleus ROI (*yellow*) and the FISH signal ROIs (in *green*, *red* and *blue*)

In order to localize FISH signals and measure relative distances between them, cells were processed for FISH and imaged in high-throughput mode (see "Materials and methods"). For image analysis, we adapted a previously described custom image analysis script [18]. Briefly, image stacks of each channel for each field of view were maximally projected in two dimensions and nuclei were segmented based on the DAPI (4',6-diamidino-2-phenylindole; fluorescent nuclear DNA stain) channel (Fig. 2a, b). Irregularly shaped segmented nuclei, often due to segmentation errors, and nuclei touching the image border, were excluded from further analysis. The nucleus region of interest (ROI) was then used as the search region for FISH spot detection in the Green, Red and FarRed channels (Figure 2c–j). To determine the relative position of FISH signals, center-to-center Euclidean distances between each Red FISH signal and all of the Green and FarRed signals in the same cell were measured (see "Materials and methods"). Output attributes of the hiBA-FISH image analysis pipeline include the number of nuclei, number of FISH spots detected per cell in each channel, and complete Red/Green and Red/FarRed distance datasets for all detected Red FISH signals (see "Materials and methods").

**Fig. 3:**
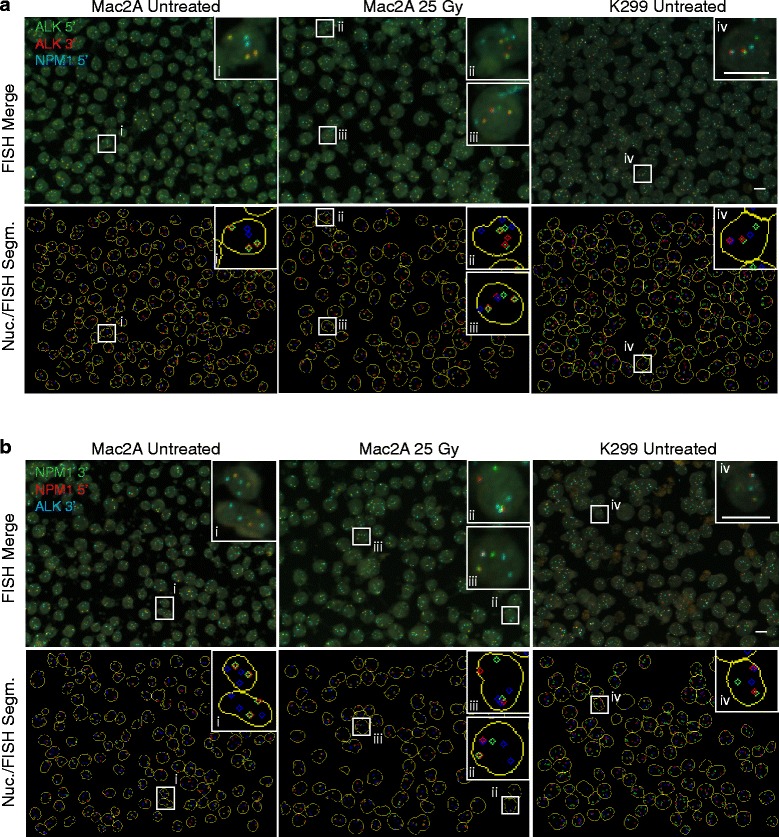
Qualitative identification of breakage and translocation events with hiBA-FISH. **a** Representative maximal projections of 40× confocal images of *NPM1-ALK* translocation-negative Mac2A cells, untreated or treated with 25 Gy of ionizing radiation, and untreated *NPM1-ALK* translocation-positive K299 cells stained with the hiBA-ALK probe set. Overlays of the three FISH probe channel images [19], the nucleus segmentation (*yellow*) and FISH spot detection (*bottom*). Magnification of an intact *ALK* allele event (*i*). Broken *ALK* allele events (*ii*). *NPM1-ALK* translocation events (*iii* and *iv*). *Scale bar* 10 μm. **b** Same as (**a**), but cells were stained with the hiBA-NPM probe set. Magnification of an intact *NPM1* allele event (*i*). Broken *NPM1* allele events (*ii*). *NPM1-ALK* translocation events ((*iii* and *iv*)). *Scale bars* 10 μm

Qualitative visual inspection of *NPM1*-*ALK* translocation-negative Mac2A and *NPM1*-*ALK* translocation-positive K299 cells [19] confirmed the predicted spatial positioning patterns for the FISH probe sets (Fig. 3). As expected, in Mac2A cells, Red and Green break-apart probe signals for both probe sets were almost exclusively in spatial proximity of each other, indicating intact *ALK* and *NPM1* alleles in the majority of cells (Fig. 3a(i) and Fig. 3b(i)). Separation of Red and Green signals indicating *ALK* or *NPM1* breakage (Fig. 3a(ii) and Fig. 3b(ii)) and concomitant spatial proximity of separated Red signals with FarRed signals indicating *NPM1*-*ALK* translocations were found in a small number of Mac2A cells after treatment of cells with 25 Gy of ionizing radiation to induce global DNA damage (Fig. 3a(iii) and Fig. 3b(iii)). As expected, almost all *NPM1*-*ALK* translocation-positive K299 cells showed separation of at least one Red and Green break-apart probe pair and spatial proximity of the separated Red signal with a FarRed signal (Fig. 3a(iv) and Fig. 3b(iv)).

### hiBA-FISH signal quantification

**Fig. 4:**
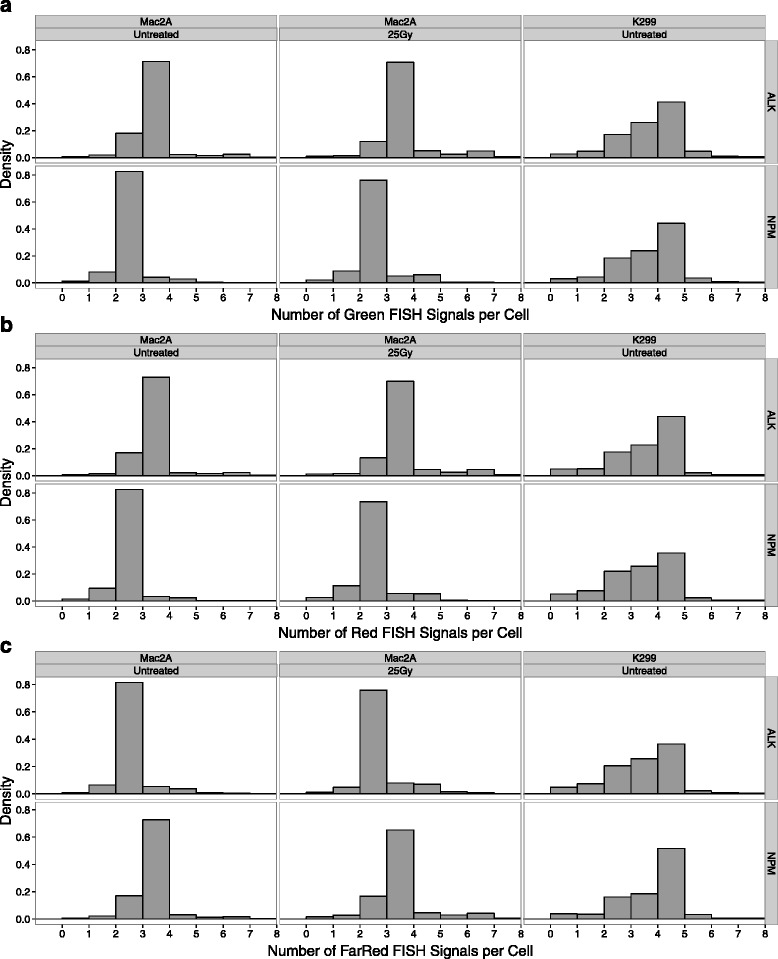
Automated hiBA-FISH signal detection metrics. **a** Histograms of the distributions of Green FISH signal number per cell as measured by automated image analysis in the indicated cell lines and experimental conditions. *ALK* hiBA-ALK probe set, *NPM* hiBA-NPM1 probe set. Bin size = one FISH signal per cell. The first bin includes cells with zero spots. **b** Same as (**a**), but for Red FISH signals. **c** Same as (**a**), but for FarRed FISH signals

Automated hiBA-FISH image analysis was used to quantify FISH signal positioning patterns and inter-signal distances in the cell population. Quantitative signal data were generated by automated analysis of more than 5000 individual *NPM1-ALK* translocation-negative Mac2A cells and ~2000 *NPM1-ALK* translocation-positive K299 cells. FISH signals were detected with greater than 99 % accuracy based on comparison of visual and automated detection of FISH signals (data not shown). In agreement with previous visual counting of FISH spots [19], most Mac2A cells had three *ALK* alleles (71.4 % using the hiBA-ALK Green probe, 72.9 % hiBA-ALK Red, 72.6 % hiBA-NPM1 FarRed; total number of nuclei 10,563 for hiBA-ALK and 8802 for hiBA-NPM1) and two *NPM1* alleles (82.7 % hiBA-NPM1 Green, 82.5 % hiBA-NPM1 Red, 81.6 % hiBA-ALK FarRed; total number of nuclei 10,563 for hiBA-ALK and 8802 for hiBA-NPM1) (Fig. 4a–c). In K299 cells, subpopulations of cells with two, three or four *NPM1* and *ALK* alleles were detected with the major subpopulation containing four alleles for both genes using both hiBA-FISH probe sets (Fig. 4a–c) [20]. Irradiation of Mac2A cells did not alter the FISH detection efficiency when compared with untreated samples (Fig. 4a–c). Altogether, these results indicate that hiBA-FISH can be used for the precise, robust and high-throughput detection of FISH signals and their spatial arrangement in interphase nuclei in multiple channels.

### Determination of FISH signal separation and proximity thresholds

**Fig. 5:**
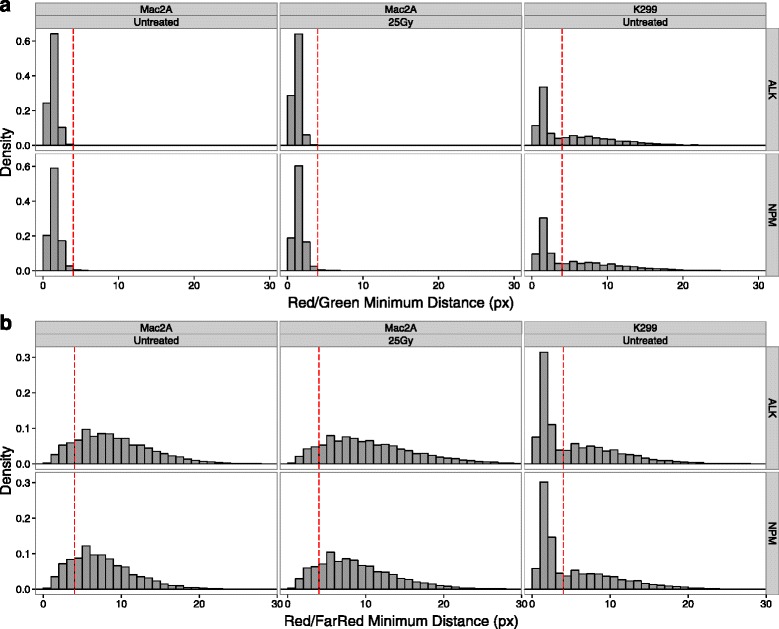
Experimental determination of a proximity threshold for FISH signals based on hiBA-FISH distances. **a** Distance distribution histograms of minimum Red/Green FISH signal distances per Red allele in the indicated cell lines and experimental conditions. *ALK* hiBA-ALK probe set, *NPM* hiBA-NPM1 probe set. One pixel = 320 nm. Bin size = one pixel. The first bin includes distances between zero and less than one pixel. The *red dashed line* represents a threshold of four pixels (1.28 μm). **b** Same as (**a**), but for minimum Red/FarRed FISH signal distances

To establish a threshold for the separation between break-apart probes, indicating chromosome breakage, we plotted the distribution of minimum Red/Green distances in non-irradiated Mac2A cells, which are not expected to contain breaks. Only distances from cells that had at least two FISH signals in both channels and the same number of Red and Green spots were considered in order to eliminate cells with missed or spurious FISH spot detection events. Using hiBA-ALK and hiBA-NPM1 probe sets, 99.8 % (total number of hiBA-ALK Red FISH signals 24,217) and 99.5 % (total number of hiBA-NPM1 Red FISH signals 14,866) of Red FISH signals were separated by four or fewer pixels (1.28 μm) from the closest Green FISH signal, respectively (Fig. 5a), with a median Red/Green distance of one pixel for both probe sets. Based on these data, we chose separation between break-apart probes by more than four pixels as an indicator of chromosome breakage. The accuracy of this threshold was validated in translocation-positive K299 cells, where 43.5 % (total number of hiBA-ALK Red FISH signals 9496) and 45.0 % (total number of hiBA-NPM1 Red FISH signals 8225) of Red FISH signals were separated by more than four pixels from the closest Green FISH signal using hiBA-ALK and hiBA-NPM1 probe sets, respectively, consistent with the presence of at least one *NPM1-ALK* translocation per cell in the vast majority of cells (Fig. 5a).

In line with a threshold of four or fewer pixels as an indicator of an intact locus, we also defined co-localization of 5’ *NPM1* and 3’ *ALK* probes in translocation events as a distance of four or fewer pixels (Fig. 5b). In *NPM1-ALK* translocation-negative Mac2A cells, 15.1 % (total number of hiBA-ALK Red FISH signals 24,217) and 20.9 % (total number of hiBA-NPM1 Red FISH signals 14,866) of 3’ *ALK* and 5’ *NPM1* pairs had distances of four or fewer pixels when detected using the hiBA-ALK or the hiBA-NPM1 probe sets, respectively. The higher percentage of proximal 5’ *NPM1* and 3’ *ALK* pairs for the hiBA-ALK probe is accounted for by the presence of three *ALK* alleles compared with only two *NPM1* alleles in Mac2A cells [19]. In contrast, in K299 cells, 54.5 % (total number of hiBA-ALK Red FISH signals 9496) and 55.9 % (total number of hiBA-NPM1 Red FISH signals 8225) of 3’ *ALK* and 5’ *NPM1* pairs were in spatial proximity (≤4 pixels) using hiBA-ALK and hiBA-NPM1 probes, respectively (Fig. 5b), in accordance with the observation that approximately half of *ALK* and *NPM1* alleles are translocated in these cells [21].

### Quantification of chromosome breaks

**Fig. 6:**
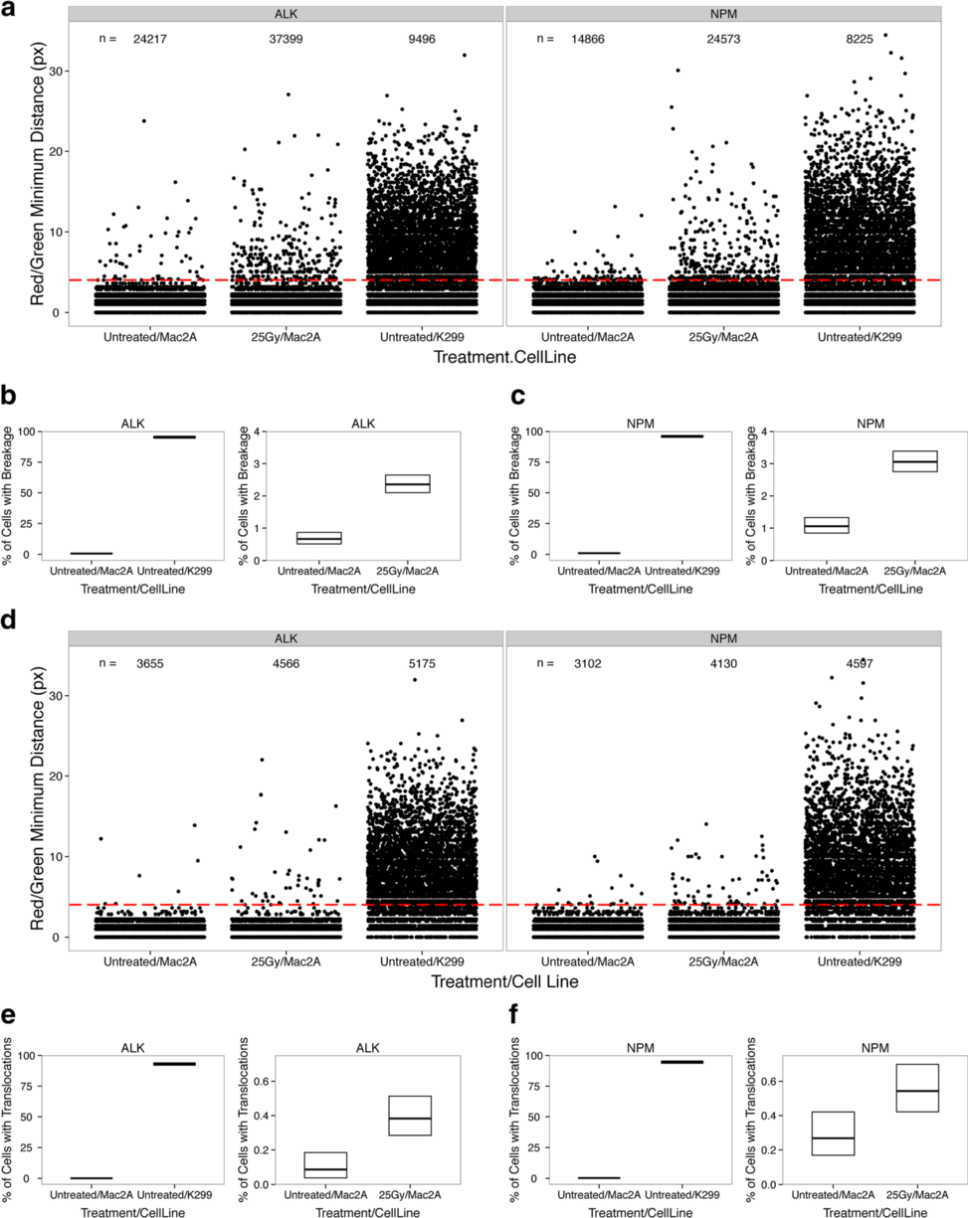
Automated determination of chromosome breaks and translocations by hiBA-FISH. **a** Jitter plot of the minimum Red/Green FISH per Red allele signal distances in the indicated cell lines and experimental conditions. *ALK* hiBA-ALK probe set, *NPM* hiBA-NPM1 set. One pixel = 320 nm. Each dot represents a measured Red/Green distance. The number of Red/Green distances plotted for each single experimental condition is indicated. Breakage events have a Red/Green minimum distance of more than four pixels (1.28 μm, *red dashed line*). **b** Crossbar plot indicates the frequency of cells with at least one breakage event (*middle line*) and its relative 95 % CI (from top to bottom line) expressed as percentages for the indicated cell lines and treatments as measured with the hiBA-ALK probe set for the datasets shown in (**a**). The same frequency values relative to *Untreated/Mac2A* cells were plotted in both the left and right panels for comparison purposes. **c** Same as (**b**), but for the hiBA-NPM1 set. **d** Jitter plot of the subset of Red FISH signals in proximity of a FarRed signal (Red/FarRed minimum distance ≤4 pixels). Translocation events have a Red/Green minimum distance of more than four pixels (*red dashed line*). Each dot represents a Red/Green distance. The number of Red/Green distances plotted for each single experimental condition is indicated. **e** Crossbar plot but for the frequency of cells with at least one *NPM1-ALK* translocation event as measured by the hiBA-ALK probe set. The same frequency values relative to *Untreated/Mac2A* cells are plotted in both the left and right panels for comparison purposes. **f** Same as (**e**), but for the hiBA-NPM1 probe set

Using the thresholds determined above, we defined an *ALK* or *NPM1* breakage event as separation of the break-apart probes by more than four pixels. As predicted, an overwhelming majority of control K299 cells possessed at least one DNA break event in the *ALK* gene (hiBA-ALK, 2571/2695 nuclei, 95.4 %, 95 % confidence interval (CI) 94.5–96.1 %) or in the *NPM1* gene (hiBA-NPM1, 2352/2448 nuclei, 96.1 %, 95 % CI 95.2–96.8 %) per cell (Fig. 6a–c). In contrast, in untreated Mac2A cells, where *ALK* and *NPM1* breakage is not expected, the percentage of cells carrying at least one *ALK* break or one *NPM1* break was 0.66 % (hiBA-ALK, 53/7984 nuclei, 95 % CI 0.50–0.87 %) and 1.05 % (hiBA-NPM, 75/7089 nuclei, 95 % CI 0.84–1.32 %), respectively (Fig. 6a–c).

To determine the sensitivity of hiBA-FISH, we induced chromosome breaks and translocation by irradiation of Mac2A cells with 25 Gy, generating 500–750 DNA breaks per diploid genome [22]. Considering that the break-apart probes are separated by ~100 kb of DNA, a breakage event in the probed *ALK* and *NPM1* region is expected in ~1 out of 50 cells. In line with this estimate, when compared with untreated cells the percentage of detected chromosome breaks upon irradiation increased 3.6-fold from 0.66 % (53/7984 nuclei, 95 % CI 0.50–0.86 %) to 2.36 % (277/11,753 nuclei; 95 % CI 2.10–2.65 %; Fisher’s exact test *p* value < 2.2e-16) for the hiBA-ALK probe (Fig. 6b) and 2.9-fold from 1.05 % (75/7089 nuclei, 95 % CI 0.84–1.32 %) to 3.05 % (343/11,230 nuclei; 95 % CI 2.75–3.39 %; Fisher’s exact test *p* value < 2.2e-16) for the hiBA-NPM1 probe (Fig. 6c). These results indicate a lower boundary for reliable detection above background of separated chromosome breaks in the range of ~1 %, equivalent to 1 in 100 cells. This sensitivity range was confirmed by titration of translocation-positive K299 cells against increasing proportions of translocation-negative Mac2A cells (Fig. S1a in Additional file 1). We conclude that hiBA-FISH is capable of detecting chromosome breakage on a per cell basis with high sensitivity.

### Sensitive detection of rare *NPM1*-*ALK* translocations

Finally, we analyzed the percentage of cells carrying at least one translocation event, defined as separation of the break-apart probe by more than four pixels with simultaneous proximity of 5’ *NPM1* and *3’ ALK* within a distance of four or fewer pixels. hiBA-FISH identified at least one *NPM1-ALK* translocation event in 93.1 % (2508/2695 nuclei, 95 % CI 92.0–94.0 %) of translocation-positive K299 cells using the hiBA-ALK probe set and in 94.6 % (2316/2448 nuclei, 95 % CI 93.6–95.4 %) using the hiBA-NPM probe set (Fig. 6d–f). This puts the false negative rate for the detection of translocations by hiBA-FISH between 5 % and 10 % assuming that the K299 cells are homogeneously *NPM1-ALK* positive. Importantly, a statistically significant difference between the percentage of *NPM1-ALK* translocations in untreated Mac2A cells and irradiated Mac2A cells was measured using both hiBA-FISH probe sets (Fig. 6e, f). Upon irradiation of Mac2A cells, the percentage of cells carrying at least one *NPM1-ALK* translocation increased 4.3-fold from 0.088 % (7/7984 nuclei; 95 % CI 0.038–0.18 %) to 0.38 % (45/11,753 nuclei; 95 % CI 0.29–0.51 %; Fisher’s exact test *p* value = 4.67e-5) as measured by the hiBA-ALK probe set (Fig. 6e); and twofold from 0.27 % (19/7089 nuclei; 95 % CI 0.17–0.42 %) to 0.54 % (61/11,230 nuclei; 95 % CI 0.42–0.70 %; Fisher’s exact test *p* value = 0.0056) for the hiBA-NPM1 probe set (Fig. 6f). Titration experiments confirm the reliable detection limit of *NPM1-ALK* translocated cells to ~0.3 %, or approximately 1 in 300 cells (Fig. S1b in Additional file 1).

Taken together, these results confirm that hiBA-FISH, through a combination of high-throughput imaging and single cell analysis of FISH signals, is a sensitive method for the detection and quantitative measurement of low-frequency breakage and translocation events in interphase cells.

## Discussion

Here, we describe an unbiased, quantitative method for the detection of rare chromosome breaks and translocations in interphase cells with high sensitivity. hiBA-FISH is based on the high-throughput measurement of the spatial separation of break-apart FISH probes and analysis of large distance distributions datasets. The method also allows for the visualization of individual, allele-specific breakage and translocation events and generates single-cell data statistics across large cell populations.

Break-apart FISH offers several advantages over conventional FISH. First, probes can be easily and rapidly designed to flank virtually any region of the genome with intervening distances ranging from a few to several hundred kilobases. In most cases, existing BAC probes, often commercially available, can be used for detection, or genomic DNA clones can be generated for regions which are not covered by available BACs. This allows, design of break-apart probes that flank chromosome breakpoint sites in non-coding regulatory regions, for example, those involved in many B- and T-cell translocations and have proven difficult to detect by FISH methods using probes targeted to the fusion RNAs [23, 24]. Second, break-apart FISH probes can be used with multiple translocation partners to determine the relative frequency of each translocation within a population of cells. For example, the 5’ *NPM1* probe in our hiBA-ALK probe mix could be substituted with any of several known, or hypothesized, *ALK* fusion partners [25]. Third, the use of reciprocal break-apart probe sets for each translocation partner, as shown here for the hiBA-ALK and hiBA-NPM1 probe sets, increases the accuracy of breakage and translocation detection. Finally, in addition to quantification of chromosome breaks and translocations, hiBA-FISH provides information on allele copy number and spatial positioning of genome regions in intact nuclei via counting of the number of FISH signals and measurement of FISH signal distances. Importantly, hiBA-FISH, unlike PCR methods, can be used to quantitatively determine the breakage frequency of a genomic region of interest, including large regions on the order of several hundred kilobases. In addition, the ability to capture all translocations in a large genome region makes hiBA-FISH suitable for detection of translocations without the requirement to map the precise translocation breakpoints or fusion product. The method is thus useful in basic research applications to probe translocation frequency of genome regions of interest and may be clinically applicable for the facile detection of unknown translocations in target genome regions.

Due to its high-throughput nature, hiBA-FISH is a highly sensitive method and is suited to analyze several thousands of cells per experimental condition, in contrast to most FISH approaches that rely on visual inspection of relatively small sample numbers and dual-fusion FISH probes which require metaphase spreads [7, 16]. The combination of break-apart probes with a third probe to detect a potential translocation partner generates a highly specific and sensitive detection system, since it requires a concomitant separation and a spatial proximity event to define a translocation. Imaging of thousands of cells per sample ensures a precise estimation of the frequency of these events. For the hiBA-ALK probe set, we detected here at least one *ALK* break in ~100 irradiated Mac2A cells and at least one *NPM1-ALK* translocation in ~300 irradiated Mac2A cells.

hiBA-FISH is a versatile experimental tool to probe the effect of biological, chemical, or physical treatments on chromosome breakage and translocation formation. While we demonstrate here hiBA-FISH in suspension cells plated on poly-D-lysine-coated coverslips, hiBA-FISH should be equally applicable to adherent cells grown directly on coverslips. In addition, hiBA-FISH can be scaled up considerably to assess multiple treatment conditions and probe combinations in the same experiment by use of multi-well plates (e.g., 96- or 384-well formats).

DNA FISH, using both two-color fusion and break-apart probes, is an established tool in the clinical setting in the diagnosis and monitoring of patients with chromosome translocations. While RT-PCR remains the gold standard for detecting known gene fusions, FISH is commonly used as a complementary cytological method to validate RT-PCR results or when one of the translocation partners is highly variable. In addition, DNA FISH is the method of choice when RT-PCR primer sets are not available for a given fusion, when a fusion involves a non-coding region, or when there is considerable breakpoint heterogeneity [7, 10]. Several FDA-approved break-apart probe sets are already available to pathologists for application to clinical samples. However, break-apart FISH is currently limited to manual or semi-automated analysis and is thus only useful for detection of translocations that occur with high frequency in a cell population. hiBA-FISH overcomes this limitation and may therefore be a useful complementary tool in diagnostics to detect rare breakage and translocation events in highly heterogeneous samples. A particularly suitable application may be the cytogenetic detection of minimal residual disease in cell populations following treatment regimes. Finally, as HTI instrumentation and image analysis tools evolve, we envision that hiBA-FISH will be applicable to tissue samples and analysis of clinical biopsy samples from solid organs.

## Conclusions

We describe hiBA-FISH, an optical method for the detection of chromosome breaks and translocations. The method is based on the use of break-apart FISH probes and their application to high-throughput imaging. We demonstrate the sensitive detection of chromosome breaks and translocations in a clinically relevant chromosome breakpoint region. The method has basic research applications and potential for clinical use in diagnostics and discovery.

## Materials and methods

### Cell culture

Translocation-positive ALCL K299 and translocation-negative ALCL Mac2A cell lines provided by Dr S. Mathas (Charite-Berlin) were maintained in RPMI-1640 at 37 °C and 5 % CO_2,_ 10 % fetal bovine serum (Atlanta Biologicals), 2 mM L-glutamine, 100 U ml^−1^ penicillin and 100 μg ml^−1^ streptomycin.

### Irradiation

DNA breaks and translocations were generated by irradiation of cells using a Cesium Mark-1 irradiator at a dose of 25 Gy as previously described [19].

### FISH

Three-dimensional FISH probes were generated from BACs (BACPAC Resources Center) by direct labeling by nick translation with fluorescently labeled dUTPs (Chromatide AlexaFluor 488-5-dUTP and 568-5-dUTP from Life Technologies; Cy5 dUTP from Fisher Scientific) using a nick translation kit (Abbott Molecular). The hiBA-ALK probe set was generated from RP11-119L19 (*ALK* 5’ break-apart, Alexa488), RP11-100C1 (*ALK* 3’ break-apart, Alexa568), RP11-1072I20 (*NPM1* 5’, Cy5), the hiBA-NPM1 probe set from RP11-1072I20 (*NPM1* 5’ break-apart, Alexa568), RP11-145P20 (*NPM1* 3’ break-apart, Alexa488), RP11-100C1 (*ALK* 3’, Cy5). The *ALK* break-apart probes were located 32 kb upstream and 65 kb downstream of the *ALK* breakpoint in intron 19 (Fig. 1c). The *NPM1* break-apart probes were located 55 kb upstream and 107 kb downstream of the known *NPM1* breakpoint in intron 4 (Fig. 1c). The sequence specificity of all probes was verified by PCR.

For 3D FISH, cells were plated on glass poly-D-lysine coated coverslips (22 × 22 mm, 170 μm thick, Neuvitro) in a 24-well (one million cells/well), plates spun at 170 g for 5 minutes, followed by incubation at 37 °C, 30 minutes. After fixation in 4 % paraformaldehyde/phosphate-buffered saline (PBS) (15 minutes), cells were permeabilized (20 minutes in 0.5 % saponin (Sigma Aldrich)/0.5 % Triton X-100/PBS) and incubated in 0.1 N HCl (15 minutes) with PBS washes between steps. After washing in 2× SSC wash, cells were incubated in 50 % formamide/2× SSC buffer (30 minutes). Probe mix (80 ng of each probe, 3 μg COT1 DNA (Roche) and 20 μg tRNA (Ambion)) were ethanol precipitated and resuspended in 7.5 μl hybridization buffer (10 % dextran sulfate, 50 % formamide, 2× SSC, and 1 % Tween-20) and added to each coverslip. Denaturation of cells and probes was at 85 °C for 5 minutes and hybridization in a humidified chamber overnight at 37 °C. Excess probe was removed by three 5-minute washes in 1× SSC at 45 °C, followed by three 5-minute washes in 0.1× SSC at 45°C. Coverslips were mounted on glass slides (Tekdon, Myakka City, FL, USA) in DAPI-containing Vectashield (Vector, Burlingame, CA, USA).

### High-throughput imaging

Imaging of mounted coverslips was performed on an Opera QEHS High-throughput confocal microscope (PerkinElmer, Waltham, MA, USA) running Opera 1.8.1 or Opera 2.0.1 software, equipped with a slide holder adaptor using a Planar Apochromatic 40× water immersion lens (Olympus, NA = 0.9) and 1.3 MegaPixel CCD cameras with pixel binning of 2, corresponding to a pixel size of 320 nm. DAPI, Alexa488, Alexa568 and Cy5 images were sequentially acquired in more than 50 fields per coverslips in separate exposures using seven z-planes (1.5 μm apart). Typically, at least 5000 Mac2A cells and at least 2000 K299 cells were imaged per experimental condition. The full raw image datasets are available from the Dryad Digital Repository [26].

### Automated image analysis

Image analysis was performed using a modified version of a previously custom script running on Acapella 2.6 (PerkinElmer, Waltham, MA, USA) [18]. In brief, nuclei were segmented based on the DAPI signal in maximally projected images and nuclear area and roundness were calculated. Non-nuclear objects likely representing nuclear debris and/or nuclear segmentation errors were eliminated by setting nuclear area and roundness filters. Nuclear ROIs were then used to constrain the sequential detection of FISH signals using a previously described spot detection algorithm [18]. Center-to-center, Euclidean 2D distances between all the possible combinations of FISH signals (Alexa568/Alexa488, Red/Green; Alexa568/Cy5, Red/FarRed) in a single nucleus were determined from segmented FISH signal ROIs. All single-cell and single-spot-distance level data were exported as independent text files. The Acapella script and parameter files are available from the Dryad Digital Repository [26].

### Data analysis

Statistical data analysis was performed using R (version 3.2.0) [27] and RStudio [28]. Text files containing the single-cell and single-distance level were read in batch and concatenated. Experimental metadata (cell line, ionizing radiation (IR) treatment, transfected construct, experiment name, FISH probe mix) were parsed from file names. The number of detected FISH signals per nucleus in each channel was joined to the single distance-level dataset by using common indexes in the two datasets. Possible FISH staining and image analysis-based FISH signal detection artifacts were excluded by keeping for downstream analysis only nuclei containing a) at least two signals in the Green, Red and FarRed channels and b) an equal number of Green and Red FISH signals. Minimum Red/Green and Red/FarRed distances were calculated on a per Red allele basis. The minimum Red/Green and Red/FarRed distances were then joined using common indexes identifying individual Red FISH signals. The FISH signal distance proximity threshold was determined to be four pixels (1.28 μm) based on measurement of Red/Green distances in unirradiated Mac2A cells. A chromosome breakage event was defined as a FISH Red signal that had a corresponding minimum Red/Green distance of more than four pixels. A chromosome translocation event was defined as a Red FISH signal that concomitantly had a corresponding minimum Red/Green distance of more than four pixels and a minimum Red/FarRed distance of four or fewer pixels. Nuclei that possessed at least one breakage or translocation were labeled as positive for the respective event class. The modified Wald method [29] was used to determine proportion confidence intervals shown in Fig. 6 and in Additional file 1. The Fisher’s exact test for count data was used for pairwise comparison of translocation or breakage event percentages between untreated and irradiated Mac2A cells. The original single-cell datasets, single-spot distance datasets, and R analysis script .rmd files are available from the Dryad Digital Repository [26].

### Data availability

A supplementary dataset is available from the Dryad Digital Repository [26]. This contains the original .flex image files originated by the PerkinElmer Opera QEHS high-throughput microscope (using either Opera 1.8.1 or Opera 2.0.1), the PerkinElmer Acapella script and parameter files used for image analysis (Acapella 2.6), the .txt single-object level image analysis results files generated by Acapella, the .Rmd files containing the R code used for the single-distance level analysis, and the .html output file generated by "knitting" the .Rmd file. The contents of the supplementary dataset are as follows:

1. Acapella_Scripts_Parameters.zip: This zipped file contains the Acapella script used to analyze all the image datasets and the Acapella parameter files for each analysis session. The parameter files are named according to the relative image dataset analyzed.
2. BC_140713_K299_K299_ALK_UN.zip: This zipped file contains the image dataset relative to untreated K299 cells stained with the hiBA-ALK probe set. This dataset was used to generate Figs. 3, 4, 5 and 6.
3. BC_140713_K299_K299_NPM_UN.zip: This zipped file contains the image dataset relative to untreated K299 cells stained with the hiBA-NPM1 probe set. This dataset was used to generate Figs. 3, 4, 5 and 6.
4. BC_140815_M2A_GFP_ALK_IR.zip: This zipped file contains the image dataset relative to irradiated (25 Gy) Mac2A cells stained with the hiBA-ALK probe set. This dataset was used to generate Figs. 3, 4, 5 and 6.
5. BC_140815_M2A_GFP_NPM_IR.zip: This zipped file contains the image dataset relative to irradiated (25 Gy) Mac2A cells stained with the hiBA-NPM1 probe set. This dataset was used to generate Figs. 3, 4, 5 and 6.
6. BC_140819_M2A_GFP_ALK_UN.zip: This zipped file contains the image dataset relative to untreated Mac2A cells stained with the hiBA-ALK probe set. This dataset was used to generate Figs. 3, 4, 5 and 6.
7. BC_140819_M2A_GFP_NPM_UN.zip: This zipped file contains the image dataset relative to untreated Mac2A cells stained with the hiBA-NPM1 probe set. This dataset was used to generate Figs. 3, 4, 5 and 6.
8. BC_150527_K299_HUNDRED_ALK_UN.zip: This zipped file contains the image dataset relative to 100 % untreated K299 cells stained with the hiBA-ALK probe set. This dataset was used to generate Additional file 1.
9. BC_150527_K299_ONE_ALK_UN.zip: This zipped file contains the image dataset relative to 1 % untreated K299 cells stained with the hiBA-ALK probe set. This dataset was used to generate Additional file 1.
10. BC_150527_K299_POINTFIVE_ALK_UN.zip: This zipped file contains the image dataset relative to 0.5 % untreated K299 cells stained with the hiBA-ALK probe set. This dataset was used to generate Additional file 1.
11. BC_150527_K299_POINTONE_ALK_UN.zip: This zipped file contains the image dataset relative to 0.1 % untreated K299 cells stained with the hiBA-ALK probe set. This dataset was used to generate Additional file 1.
12. BC_150527_K299_TEN_ALK_UN.zip: This zipped file contains the image dataset relative to 10 % untreated K299 cells stained with the hiBA-ALK probe set. This dataset was used to generate Additional file 1.
13. BC_150527_K299_ZERO_ALK_UN.zip: This zipped file contains the image dataset relative to 0 % untreated K299 cells (100 % Mac2A cells) stained with the hiBA-ALK probe set. This dataset was used to generate Additional file 1.
14. R_Analysis_Fig3_6.zip: This zipped file contains the .txt single-object level image analysis results files generated by Acapella, the .Rmd R script used for the analysis and the .html R output file. This .Rmd R script was used to generate Figs. 3, 4, 5 and 6.
15. R_Analysis_FigS1.zip: This zipped file contains the .txt single-object level image analysis results files generated by Acapella, the .Rmd R script used for the analysis and the .html R output file. This .Rmd R script was used to generate Additional file 1.

## Additional file

Additional file 1:
**Titration of translocation-positive K299 cells Fig. S1 a** **K299 cells were serially diluted with increasing quantities of Mac2A cells and hiBA-FISH was performed using the hiBA-FISH ALK probe set.** The *middle line* in the *crossbars* indicates the percentage of cells with at least one *ALK* breakage event for each K299 serial dilution point. The *upper* and *lower lines* in the crossbar define the limits of the 95 % confidence interval (CI) for the percentage. **b** Same as (**a**), but the *middle line* in the *crossbar* represents the percentage of cells with at least one *NPM1-ALK* translocation event. The *upper* and *lower lines* in the *crossbar* define the limits of the 95 % confidence interval (CI) for the percentage. (PDF 763 kb)

## Abbreviations

3D: three-dimensional
ALCL: anaplastic large cell lymphoma
BAC: bacterial artificial chromosome
bp: base pair
CI: confidence interval
DAPI: 4',6-diamidino-2-phenylindole
FISH: fluorescence in situ hybridization
Gy: Grays
hiBA-FISH: high-throughput break-apart fluorescence in situ hybridization
HTI: high-throughput imaging
kb: kilobases
PBS: phosphate-buffered saline
PCR: polymerase chain reaction
ROI: region of interest
RT-PCR: reverse transcriptase polymerase chain reaction
