# Supplementary material for: Quantitative detection of rare interphase chromosome breaks and translocations by high-throughput imaging
Source: Genome Biol. 2015 Aug 3;16(1):146. doi: 10.1186/s13059-015-0718-x (PMC4531802; doi:10.1186/s13059-015-0718-x)
Supplement: Additional file 1: — Titration of translocation-positive K299 cells Fig. S1 a K299 cells were serially diluted with increasing quantities of Mac2A cells and hiBA-FISH was performed using the hiBA-FISH ALK probe set. The middle line in the crossbars indicates the percentage of cells with at least one ALK breakage event for each K299 serial dilution point. The upper and lower lines in the crossbar define the limits of the 95 % confidence interval (CI) for the percentage. b Same as (a), but the middle line in the crossbar represents the percentage of cells with at least one NPM1-ALK translocation event. The upper and lower lines in the crossbar define the limits of the 95 % confidence interval (CI) for the percentage. (PDF 763 kb) [file 13059_2015_718_MOESM1_ESM.pdf]

A

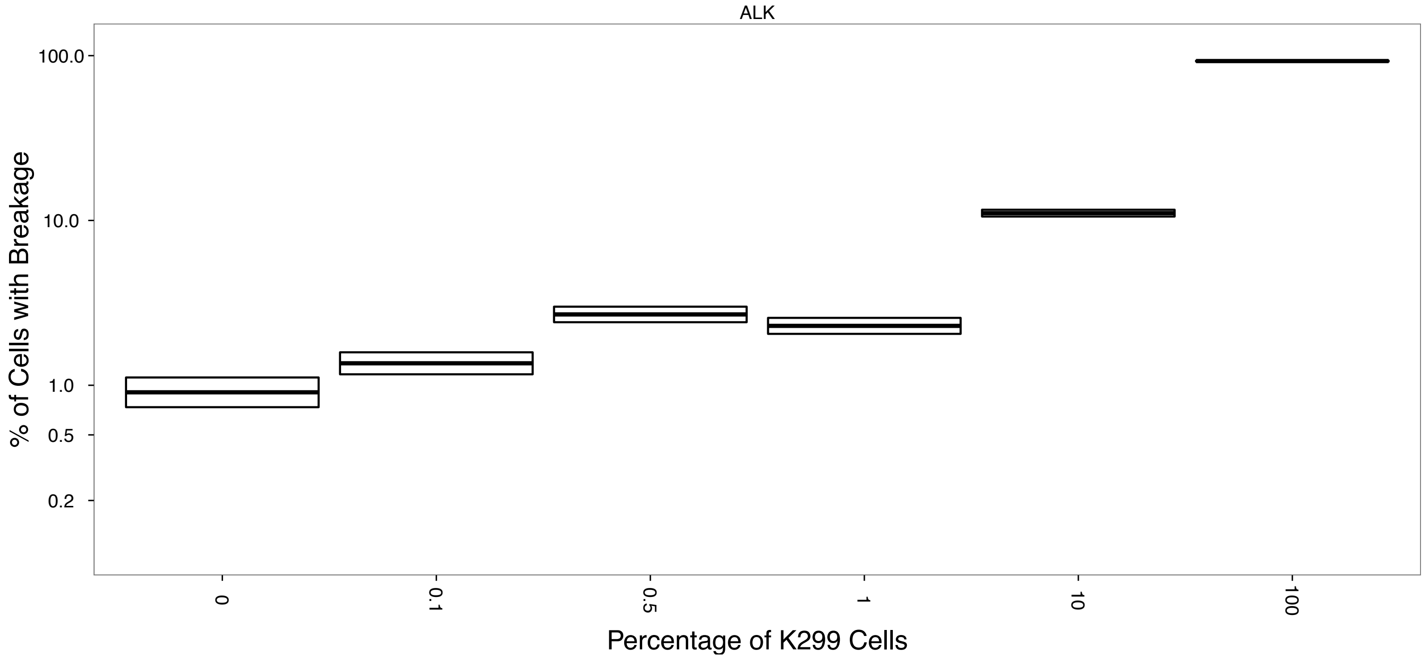

B

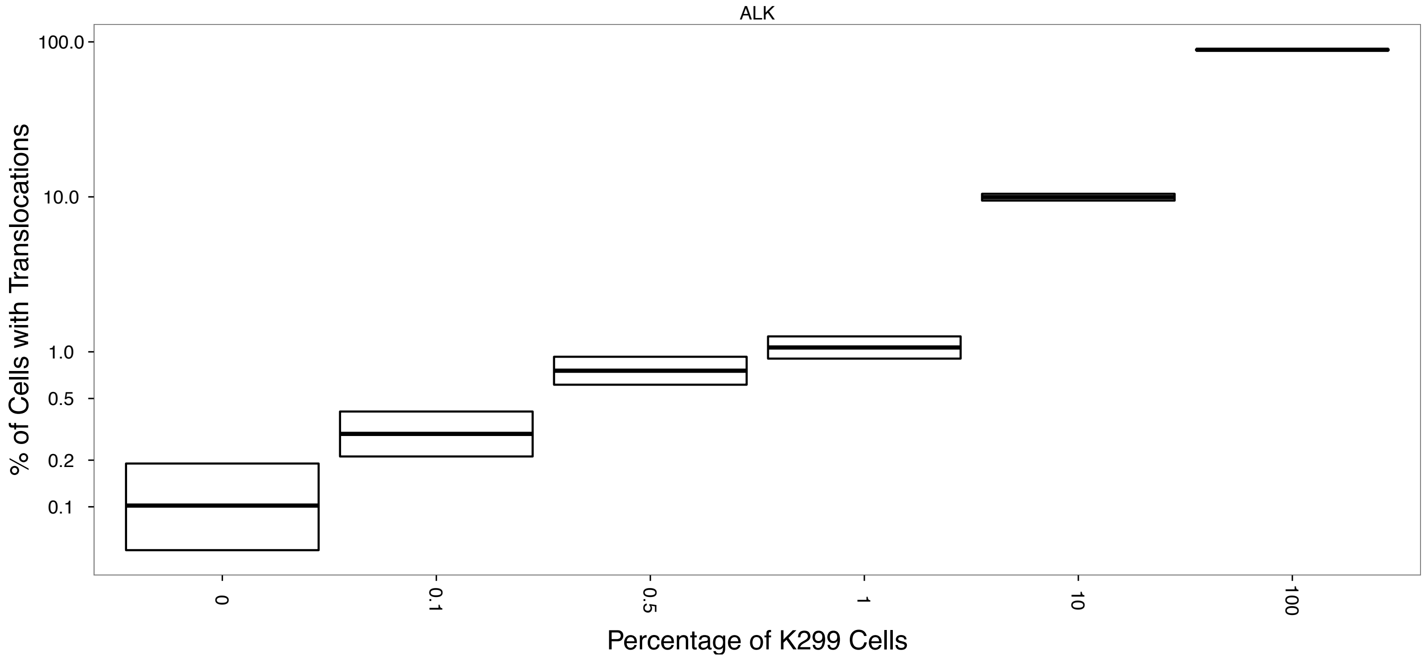

Fig. S1. A) K299 cells were serially diluted with increasing quantities of Mac2A cells and hiBA-FISH was performed using the hiBA-FISH ALK probe set. The middle line in the crossbars indicates the percentage of cells with at least one *ALK* breakage event for each K299 serial dilution point. The upper and lower lines in the crossbar define the limits of the 95% confidence interval (CI) for the percentage. **B)** Same as A), but the middle line in the crossbar represents the percentage of cells with at least one *NPM1-ALK* translocation event. The upper and lower lines in the crossbar define the limits of the 95% confidence interval (CI) for the percentage.
